# Small Heat-Shock Proteins, IbpAB, Protect Non-Pathogenic *Escherichia coli* from Killing by Macrophage-Derived Reactive Oxygen Species

**DOI:** 10.1371/journal.pone.0120249

**Published:** 2015-03-23

**Authors:** Laura Goeser, Ting-Jia Fan, Sandrine Tchaptchet, Nikolas Stasulli, William E. Goldman, R. Balfour Sartor, Jonathan J. Hansen

**Affiliations:** 1 Center for Gastrointestinal Biology and Disease, University of North Carolina at Chapel Hill, Chapel Hill, North Carolina, United States of America; 2 Department of Medicine, Division of Gastroenterology and Hepatology, University of North Carolina at Chapel Hill, Chapel Hill, North Carolina, United States of America; 3 Department of Microbiology and Immunology, University of North Carolina at Chapel Hill, Chapel Hill, North Carolina, United States of America; U. S. Salinity Lab, UNITED STATES

## Abstract

Many intracellular bacterial pathogens possess virulence factors that prevent detection and killing by macrophages. However, similar virulence factors in non-pathogenic bacteria are less well-characterized and may contribute to the pathogenesis of chronic inflammatory conditions such as Crohn’s disease. We hypothesize that the small heat shock proteins IbpAB, which have previously been shown to reduce oxidative damage to proteins in vitro and be upregulated in luminal non-pathogenic Escherichia strain NC101 during experimental colitis in vivo, protect commensal *E*. *coli* from killing by macrophage-derived reactive oxygen species (ROS). Using real-time PCR, we measured *ibpAB* expression in commensal *E*. *coli* NC101 within wild-type (wt) and ROS-deficient (*gp91phox^-/-^*) macrophages and in NC101 treated with the ROS generator paraquat. We also quantified survival of NC101 and isogenic mutants in wt and *gp91phox^-/-^* macrophages using gentamicin protection assays. Similar assays were performed using a pathogenic *E*. *coli* strain O157:H7. We show that non-pathogenic *E*. *coli* NC101inside macrophages upregulate *ibpAB* within 2 hrs of phagocytosis in a ROS-dependent manner and that *ibpAB* protect *E*. *coli* from killing by macrophage-derived ROS. Moreover, we demonstrate that ROS-induced *ibpAB* expression is mediated by the small *E*. *coli* regulatory RNA, *oxyS*. *IbpAB* are not upregulated in pathogenic *E*. *coli* O157:H7 and do not affect its survival within macrophages. Together, these findings indicate that *ibpAB* may be novel virulence factors for certain non-pathogenic *E*. *coli* strains.

## Introduction

Pathogenic *Escherichia coli* are a major source of morbidity, and less-commonly mortality, due to infections of the urinary tract, intestinal tract, and bloodstream. Most *E*. *coli* virulence factors identified to date target interactions with host intestinal epithelial cells. For instance, Esp and Nle Type III secretion system effectors from enteropathogenic (EPEC) and enterohemorrhagic (EHEC) *E*. *coli* disrupt internalization, protein secretion, NF-κB signaling, MAPK signaling, and apoptosis in eukaryotic cells[1]. Certain strains of pathogenic *E*. *coli*, including the enteroaggregative *E*. *coli*, also form biofilms in the intestine, secrete toxins that cause fluid secretion from intestinal epithelial cells, or inhibit eukaryotic protein synthesis resulting in intestinal injury[2–5].

Pathogenic *E*. *coli* that breach the intestinal mucosal barrier are phagocytosed by innate immune cells such as lamina propria macrophages and neutrophils. Some pathogenic *E*. *coli* strains have also acquired virulence genes that allow them to avoid destruction within phagocytes and thereby promote disease[6]. For example, uptake of EHEC into macrophages is associated with increased expression of Shiga toxin, and Shiga toxin enhances intra-macrophage survival through an unknown mechanism[6,7]. Likewise, expression of nitric oxide reductase in EHEC enhances their survival within macrophage phagolysosomes presumably by protecting them from reactive nitrogen species [8].

Similar to pathogenic strains of *E*. *coli*, resident intestinal (commensal) *E*. *coli* also encounter lamina propria macrophages in the intestine, especially during periods of epithelial damage and enhanced mucosal permeability in chronic inflammatory lesions associated with the inflammatory bowel diseases (IBD’s), Crohn’s disease and ulcerative colitis. IBD’s are associated with genetically-determined defective innate immune responses including disordered cytokine secretion and bacterial clearance in macrophages[9,10]. In addition IBD’s and experimental murine colitis are associated with increased numbers of luminal commensal *E*. *coli*[11]. Therefore, it is plausible that enhanced survival of *E*. *coli* in macrophages may play a role in etiopathogenesis of IBD’s. Indeed, others have shown that resident adherent- invasive *E*. *coli* are more prevalent in inflamed ileal tissue from Crohn’s disease patients compared with controls and that a specific adherent-invasive *E*. *coli* strain isolated from a human Crohn’s disease patient causes experimental colitis in susceptible hosts in vivo and survives better in macrophages in vitro compared with laboratory reference *E*. *coli* strains[12–14]. The increased survival of the adherent-invasive *E*. *coli* strain in macrophages is due in part to expression of *E*. *coli htrA*, a gene that allows *E*. *coli* to grow at elevated temperatures and defend against killing by hydrogen peroxide in vitro[15]. Genes, including *htrA*, may therefore function as virulence factors in commensal *E*. *coli* by protecting the bacteria from toxic reactive oxygen species (ROS) and/or reactive nitrogen species (RNS) found in macrophage phagolysosomes.

Similar to HtrA, the *E*. *coli* small heat shock proteins IbpA and IbpB also protect bacteria from killing by heat and oxidative stress in laboratory cultures[16–18]. The role of the *ibpAB* operon in protecting *E*. *coli* from heat damage is reinforced by evidence that *ibpAB* are upregulated in *E*. *coli* cultures in response to heat treatment[19,20]. In addition, we have previously shown that a commensal adherent-invasive murine strain of *E*. *coli* (NC101), which causes colitis in mono-colonized *Il10* ^*-/-*^ mice, increases *ibpAB* expression when present in the inflamed vs. healthy colon, possibly due to the increased concentrations of ROS/RNS in inflamed colon tissue[21–23]. However, it is unknown whether *ibpAB* are upregulated in response to ROS/RNS are important for the survival of non-pathogenic *E*. *coli* in macrophage phagolysosomes. We hypothesized that commensal *E*. *coli* upregulate *ibpAB* in response to ROS and that *ibpAB* protect *E*. *coli* from ROS-mediated killing within macrophages.

## Materials and Methods

### Bacterial Strains, Cells Lines, and Culture Conditions

The non-pathogenic murine *E*. *coli* strain NC101 was isolated as described previously[24]. *E*. *coli* strain O157:H7 was a kind gift from Dr. Ann Matthysse at UNC, Chapel Hill. *E*. *coli* were grown in Luria-Burtani (LB) broth at 37°C with shaking at 250 rpm. The J774 murine macrophage and L929 fibroblast cell lines were originally obtained from ATCC (Manassas, VA) and cultured in RPMI containing 10% fetal bovine serum (FBS), 100U/mL penicillin, 1000 μg/mL streptomycin, and 10mM glutamine in 37°C humidified incubators with 5% CO_2_. Conditioned media from L929 cells was used as a source of macrophage colony stimulating factor (M-CSF) for the production of bone marrow-derived macrophages (BMDMs) and was made as described previously[25].

The mutant *E*. *coli* NC101 strain lacking *ibpA* and *ibpB* (NC101Δ*ibpAB*) that was used in this study had been generated previously using the λ*-red* recombinase method[23,26]. We used identical methods to create a mutant *E*. *coli* O157:H7 strain that lacks *ibpA* and *ibpB* (O157:H7Δ*ibpAB*). However, since the pCP20 plasmid encoding Flp recombinase failed to induce recombination at the FRT sites in *E*. *coli* O157:H7, we used strains of NC101Δ*ibpAB* and O157:H7Δ*ibpAB* that still contained the kanamycin resistance gene. Mutant *E*. *coli* NC101 lacking *oxyS* (NC101Δ*oxyS*) was also generated using the λ*-red* recombinase method. Primers 5’GCATAGCAACGAACGATTATCCCTATCAAGCATTCTGACTGTGTAGGCTGGAGCTGCTTC and 5’ ACCGTTACTATCAGGCTCTCTTGCTGTGGGCCTGTAGAATCATATGAATATCCTCCTTAGTTCC were used to amplify the kanamycin resistance cassette from pKD4. Transformation and site-specific recombination of the PCR product into the *oxyS* locus on the *E*. *coli* NC101 chromosome followed by excision of the kanamycin resistance gene using pCP20 was performed as previously described[23,26]. Recombinant bacterial cell lines were generated in accordance with procedures outlined by the Environmental Health and Safety Department at University of North Carolina at Chapel Hill.

### Mouse Strains and Production of Bone Marrow-Derived Macrophages

Wild-type, *gp91phox* ^*-/-*^, and *Inos* ^*-/-*^ mice (all on the C57/B6 genetic background) were originally obtained from Jackson Laboratories and maintained in specific-pathogen-free conditions in Department of Lab and Animal Medicine facilities at UNC, Chapel Hill. All animal protocols were approved by the UNC-Chapel Hill Institutional Animal Care and Use Committee.

Bone marrow derived macrophages (BMDMs) were obtained similar to methods described previously[27]. Briefly, bone marrow was harvested from femurs and tibias of mice by flushing marrow cavities with sterile RPMI through a 26G needle and red cells lysed with 0.8% ammonium chloride for 5 minutes. After washing twice with RPMI containing 10% FBS, 2.5 x 10^7^ cells/plate were added to 25cm petri dishes in 50mL RPMI/10%FBS/100U/mL penicillin/ 1000 μg/mL streptomycin/25ng/mL Fungizone/10% conditioned L929 media. Three days later, 10mL of RPMI/10%FBS/100U/mL penicillin/ 1000 μg/mL streptomycin/25ng/mL Fungizone/10% conditioned L929 media was added to each plate. On day 6, adherent cells (BMDMs) were removed with TrypLE-Express (Invitrogen), counted, and plated into experimental wells.

### Gentamicin Protection Assays

Intra-macrophage bacterial survival assays were performed as described previously[14,23]. Briefly, approximately 10 mid-log phase bacteria/cell were added to 5–7.5 x 10^5^ BMDMs/well in 12-well plates in a total volume of 1mL/well RPMI/10%FBS. Plates were centrifuged at 1000xg for 10 min, incubated for 60 min at 37°C in 5% CO_2_. The end of this incubation was considered time 0. Each well was washed and treated with media containing 100μg/mL gentamicin for 60 min at 37°C in 5% CO_2_ to kill extracellular bacteria. Media was then replaced with media containing 20μg/mL gentamicin for the duration of the experiments. At the indicated times, wells were washed 4x with 1mL PBS, then incubated for 10 min at room temperature with 0.5mL of sterile water containing 1% Triton-X100 to lyse BMDMs. Viable intracellular bacteria were enumerated by counting colony forming units (CFU) in dilutions of lysates plated on LB agar. In some experiments, J774 cells were treated with 100nM bafilomycin-A1 (Sigma), an inhibitor of the vacuolar H^+^-ATPase, 60 min prior to, and during, co-incubation with bacteria.

Intra-macrophage bacterial gene expression assays were performed similarly except 6-well plates containing 2 x 10^6^ BMDMs/well or 1 x 10^6^ J774 cells/well were used, no centrifugation step was included, and time 0 was defined as the point immediately after addition of diluted bacteria to each well. At the indicated times, wells were washed as above, but instead of adding Triton-X100, 1mL/well of Bacterial RNAProtect (Qiagen) was added to the BMDMs, incubated for 5 minutes at room temperature, and then transferred to microcentrifuge tubes. After centrifugation at 10,000xg x 5 min, pellets were frozen at -20°C for future RNA isolation.

### Stimulation of Bacterial Cultures with Paraquat

Mid-log phase 10mL cultures of *E*. *coli* growing at 30°C in LB were treated for the indicated times with the indicated concentrations of the freshly-prepared superoxide generator paraquat (Sigma) dissolved in water or water control. At each time point, bacteria from a1mL aliquot of each culture were pelleted by centrifugation at 10,000 x g for 30 sec, after which 0.5mL of Bacterial RNAProtect was immediately added. After 5 min incubation at room temperature, bacteria were pelleted again and RNA was isolated as described below.

### RNA Isolation and Real-Time PCR

Bacterial RNA was isolated from cell pellets using Qiagen RNeasy Mini columns according to the manufacturer’s instructions. Purified RNA was treated with either on-column DNase treatment (Qiagen) or Baseline-Zero DNase (Epicentre) according to the manufacturer’s instructions. Complementary DNA synthesis and real-time PCR using primers for the *E*. *coli* 16S, *oxyS*, *ibpA*, and *ibpB* genes were performed as previously described[23]. Gene expression relative to the 16S rRNA bacterial housekeeping gene was calculated using the ΔΔCt method.

## Results

### *E*. *coli* upregulate *ibpAB* following phagocytosis by macrophages

**Fig 1:**
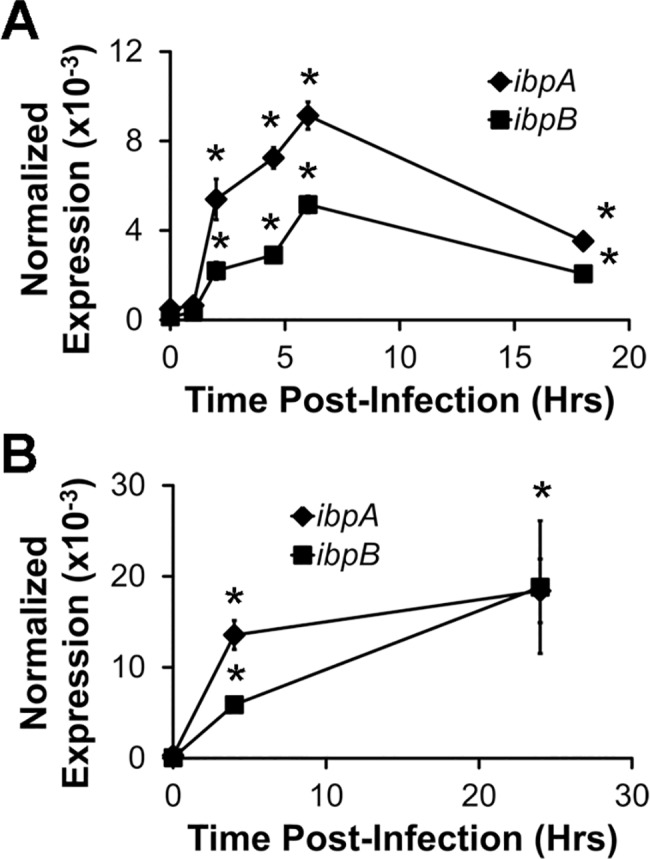
Kinetics of *E*. *coli ibpAB* upregulation during phagocytosis by macrophages. J774 macrophages (A) or BMDMs (B) were incubated with *E*. *coli* NC101. At the indicated times, *ibpA* and *ibpB* mRNA in gentamicin-resistant (i.e. intracellular) *E*. *coli* was quantified by real-time PCR. Data are presented as means ± sd (n = at least 3 wells/timepoint, *p<0.05 vs. time 0).

Since others have shown that *ibpAB* protect *E*. *coli* from oxidative damage[28,29], that *E*. *coli* upregulate other oxidative stress response genes upon phagocytosis by neutrophils[30], and that ROS are increased in macrophage phagolysosomes[31], we predicted that *E*. *coli* also upregulate *ibpAB* after phagocytosis by macrophages. To test this, we co-cultured immortalized J774 murine macrophages and murine BMDMs with the non-pathogenic murine adherent-invasive *E*. *coli* strain, NC101. At the indicated times, we quantified *ibpA* and *ibpB* mRNA in gentamicin-resistant (i.e. intracellular) *E*. *coli* using real-time PCR. We found that *E*. *coli ibpA* and *ibpB* expression increased within 2 hrs of adding bacteria and remained elevated for at least 24 hrs (Fig. 1). These data indicate that factors within macrophages induce *ibpAB* expression in *E*. *coli* relatively soon after phagocytosis.

### ROS mediate *ibpAB* expression in *E*. *coli* in cultures and macrophages

**Fig 2:**
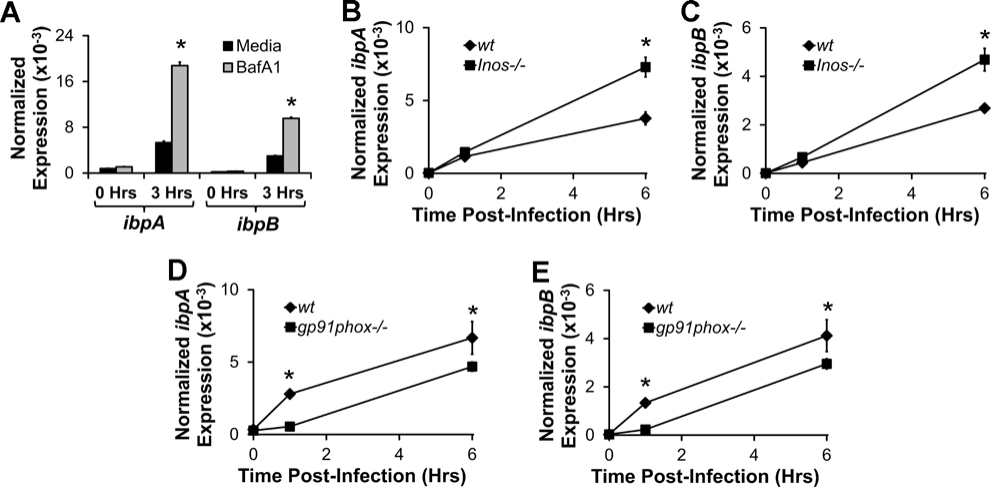
Upregulation of *ibpAB* in intracellular *E*. *coli* is partially-dependent on reactive oxygen species. A) Intracellular *E*. *coli ibpA* and *ibpB* mRNA was measured by real-time PCR in J774 macrophages that were incubated with *E*. *coli* NC101 for up to 3 hrs in the presence or absence of the vacuolar H^+^-ATPase inhibitor, bafilomycin-A1. B-E) Intracellular *E*. *coli ibpA* and *ibpB* mRNA was measured by real-time PCR in BMDMs from wt, *Inos* ^*-/-*^ (B and C) or *gp91phox* ^*-/-*^ (D and E) mice that were incubated with *E*. *coli* NC101for up to 6 hrs. Data are presented as means ± sd (n = at least 3 wells/timepoint, *p<0.05 vs. media control or wt BMDMs).

Next, we explored potential factors in macrophages that might upregulate *E*. *coli ibpAB*. To establish whether the acidic environment that exists in the macrophage phagolysosome induces *E*. *coli ibpAB*, we measured *ibpAB* expression in *E*. *coli* within J774 macrophages that had been treated with bafilomycin-A1, an inhibitor of the vacuolar H^+^-ATPase that acidifies the phagolysosome. Inhibition of vacuolar acidification did not decrease *E*. *coli ibpAB* induction within macrophages, but rather unexpectedly increased expression suggesting that the acidic environment of the phagolysosome is not responsible for upregulation of *E*. *coli ibpAB* in macrophages (Fig. 2A).

In addition to low pH, the phagolyosome also contains increased concentrations of ROS and RNS. Since *ibpAB* have been shown to protect cultured *E*. *coli* from killing by hydrogen peroxide[16], we predicted that *E*. *coli* upregulate *ibpAB* in response to phagolysosomal ROS or RNS. To test this, we incubated BMDMs from *gp91phox* ^*-/-*^ mice that have an impaired oxidative burst, *Inos* ^*-/-*^ mice that are defective in nitric oxide production, and wild-type (wt) mice with *E*. *coli* NC101 and measured *ibpAB* mRNA in intracellular *E*. *coli*. Interestingly, *ibpAB* expression in *E*. *coli* within *Inos* ^*-/-*^ BMDMs was increased relative to wt BMDMs, whereas *ibpAB* expression in *gp91phox* ^*-/-*^ BMDMs was decreased compared with wt BMDMs (Fig. 2B-E). These data suggest that ROS, but not RNS, within BMDMs are partially responsible for the induction of *ibpAB* in intra-macrophage *E*. *coli*.

**Fig 3:**
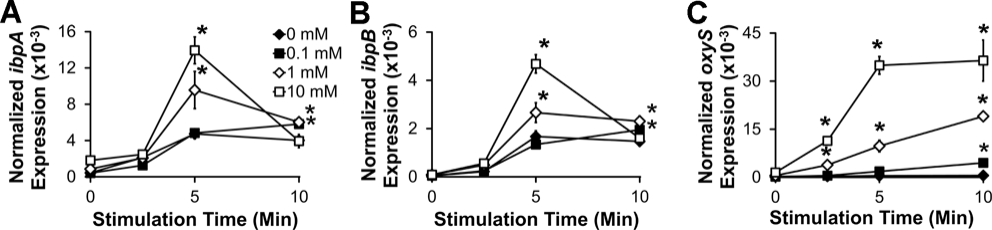
*E*. *coli* upregulate *ibpAB* in the presence of the superoxide generator, paraquat. *E*. *coli ibpA* (A), *ibpB* (B), and *oxyS* (C) mRNA was measured by real-time PCR in mid-log phase bacterial cultures that were stimulated for the indicated times with the indicated concentrations of paraquat. Data are presented as means ± sd (n = 3, *p<0.05 vs. 0mM paraquat).

To confirm that ROS enhance *ibpAB* expression in commensal *E*. *coli*, we treated mid-log phase *E*. *coli* NC101 with the superoxide generator, paraquat, for the indicated times and measured *ibpAB* expression. We detected a dose-dependent increase in *ibpAB* expression five minutes after addition of paraquat, but the degree of upregulation diminished substantially by ten minutes (Fig. 3A and B). To confirm that bacteria are sensing the presence of ROS generated by paraquat, we also measured expression of *oxyS*, a small regulatory RNA in *E*. *coli* that has previously been shown to be upregulated in response to hydrogen peroxide, control expression of several stress response genes, and protect *E*. *coli* from peroxide-induced DNA damage[32]. We observed a consistent dose- and time-dependent increase of *oxyS* mRNA in *E*. *coli* treated with paraquat (Fig. 3C). Interestingly, the *oxyS* upregulation slightly precedes *ibpAB* upregulation. These data indicate that superoxides transiently induce *ibpAB* expression in *E*. *coli* and suggest the possibility that *oxyS* mediates the superoxide-induced upregulation of *ibpAB*.

### *E*. *coli ibpAB* expression is positively controlled by the *oxyS* small regulatory RNA

**Fig 4:**
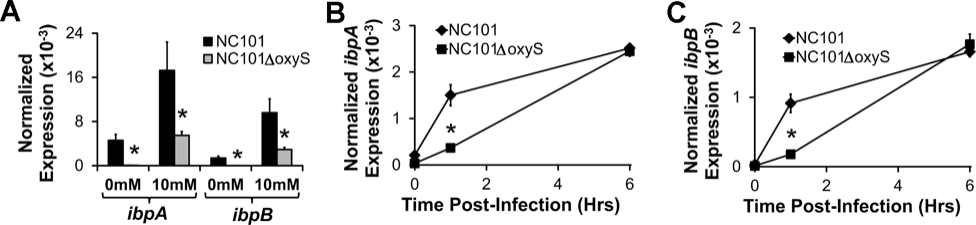
*E*. *coli ibpAB* upregulation in response to paraquat treatment and phagocytosis by macrophages is mediated in-part by *oxyS*. A) *E*. *coli ibpA* and *ibpB* mRNA was measured using real-time PCR in mid-log phase cultures of *E*. *coli* NC101 or *E*. *coli* NC101Δ*oxyS* 5 min after exposure to vehicle control (0mM) or 10mM paraquat. B and C) Intracellular *ibpA* and *ibpB* mRNA was measured using real-time PCR in wt BMDMs incubated with *E*. *coli* NC101 or *E*. *coli* NC101Δ*oxyS* for the indicated times. Data are presented as means ± sd (n = at least 3 wells/timepoint, *p<0.05 vs. *E*. *coli* NC101).

Using a reporter-gene screen, others have previously shown that *oxyS* expression up- or down-regulates 20 genes in *E*. *coli*, several of which are stress response genes[32]. However, *oxyS* has not previously been described to regulate expression of the *ibpAB* operon. Since we determined that superoxides induce *oxyS* expression shortly before *ibpAB* expression (Fig. 3), we hypothesized that *oxyS* may upregulate *ibpAB* expression. To test this, we measured *ibpAB* expression in paraquat-treated *E*. *coli* NC101 or *oxyS*-deficient *E*. *coli* (NC101Δ*oxyS*) and found that *ibpAB* expression was significantly attenuated in unstimulated as well as paraquat-stimulated NC101Δ*oxyS* (Fig. 4A). To determine whether upregulation of *ibpAB* in macrophages was also dependent on *oxyS*, we incubated BMDMs with *E*. *coli* NC101 or NC101Δ*oxyS* for the indicated times and measured *ibpAB* mRNA levels in intracellular bacteria. At one hour after the addition of bacteria, *ibpAB* mRNA was significantly lower in NC101Δ*oxyS* compared with NC101 (Fig. 4B and C). However, this difference was absent by 6 hours. Therefore, *oxyS*-dependent factors mediate *ibpAB* expression in intra-macrophage *E*. *coli* at early, but not late, stages of intracellular survival. The mechanisms by which the *oxyS* small regulatory RNA controls *ibpAB* mRNA levels are still unknown.

### Expression of *ibpAB* is associated with enhanced *E*. *coli* survival within macrophages

**Fig 5:**
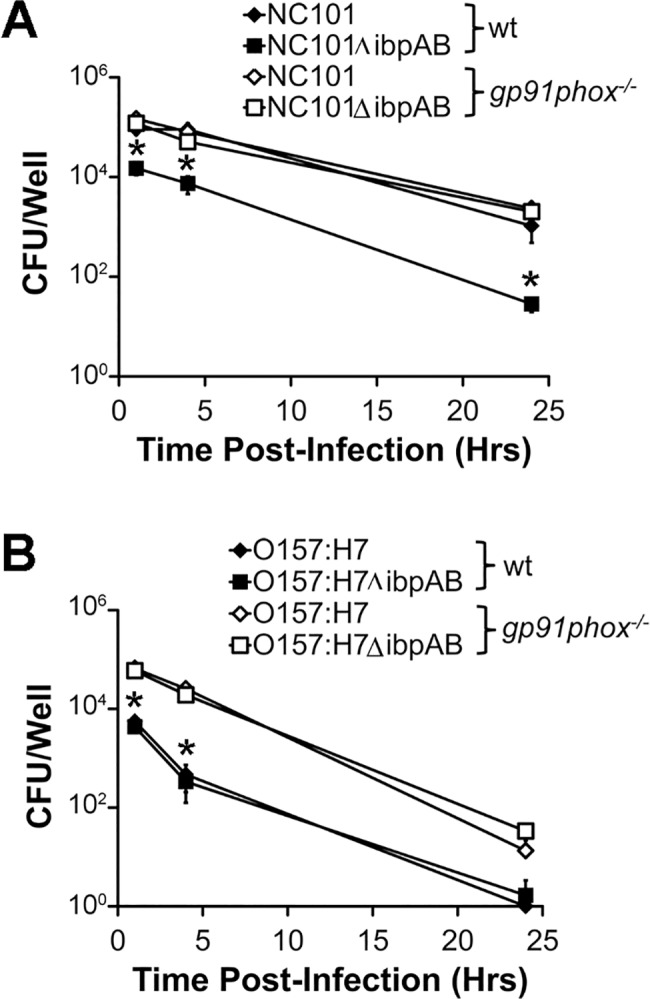
IbpAB protect commensal *E*. *coli* NC101, but not pathogenic *E*. *coli* O157:H7 from reactive oxygen species-mediated killing within macrophages. Viable, gentamicin-resistant (i.e. intracellular) *E*. *coli* were measured at the indicated timepoints after infection of wt or *gp91phox* ^*-/-*^ BMDMs with *E*. *coli* NC101 / *E*. *coli* NC101Δ*ibpAB* (A) or *E*. *coli* O157:H7 / *E*. *coli* O157:H7Δ*ibpAB* (B). Data are presented as means ± sem (n = 3–4 wells/timepoint, *p<0.05 vs. all other conditions in panel A and vs. both *E*. *coli* strains in *gp91phox* ^*-/-*^ BMDMs in panel B).

Having determined that *E*. *coli* upregulate *ibpAB* in response to ROS in culture and in macrophages, we hypothesized that *ibpAB* expression protects *E*. *coli* from killing by ROS in macrophages. In order to address this hypothesis, we incubated BMDMs from wt or *gp91phox* ^*-/-*^ mice with *E*. *coli* NC101 or *ibpAB-*deficient NC101 (NC101Δ*ibpAB*) for the indicated times and then quantified viable gentamicin-resistant (i.e. intracellular) bacteria by plating macrophage lysates on agar. At each time point examined after addition of bacteria, we detected significantly fewer intra-macrophage NC101Δ*ibpAB* vs. NC101 in wt BMDMs (Fig. 5A). However, no significant differences in intra-macrophage NC101 vs. NC101Δ*ibpAB* numbers were observed at any time point in *gp91phox* ^*-/-*^ BMDMs suggesting that *ibpAB* expression in *E*. *coli* NC101 protects intracellular *E*. *coli* from killing by macrophage-derived ROS. Interestingly, when we performed the same experiments with pathogenic *E*. *coli* O157:H7, we found that wt BMDMs kill *E*. *coli* O157:H7 more efficiently than *E*. *coli* NC101 and that *ibpAB* has no effect on intra-macrophage survival (Fig. 5B). However, unlike results observed with *E*. *coli* NC101, *gp91phox* ^*-/-*^ BMDMs kill *E*. *coli* O157:H7 less efficiently than wt BMDMs at 1 and 4 hrs post infection. Therefore, *ibpAB* protect *E*. *coli* NC101, but not *E*. *coli* O157:H7, from ROS-mediated killing in macrophages.

**Fig 6:**
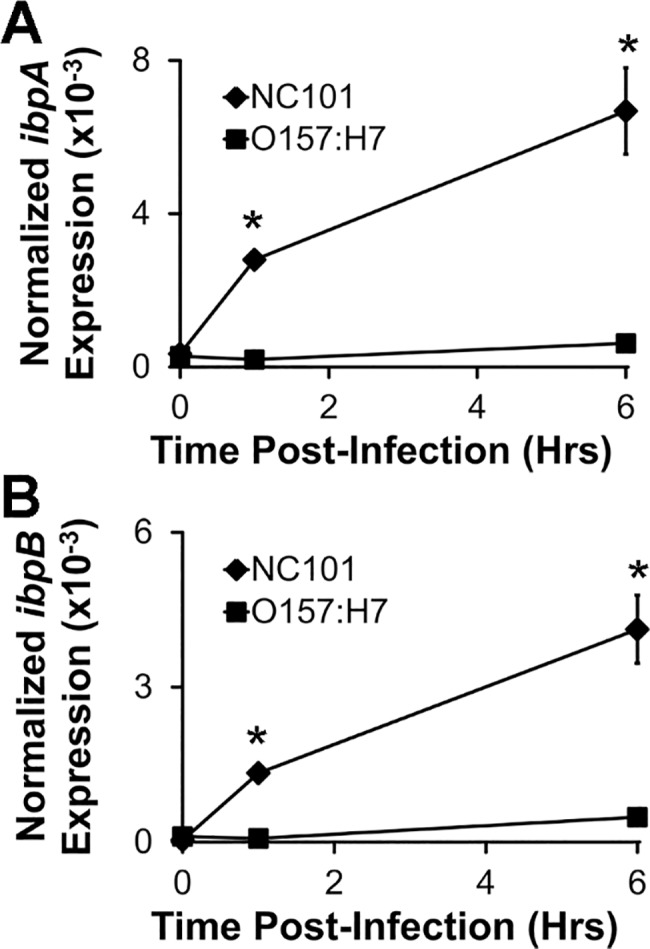
Commensal *E*. *coli* NC101, but not pathogenic *E*. *coli* O157:H7 upregulate *ibpAB* after phagocytosis by macrophages. A and B) *IbpA* and *ibpB* mRNA in intracellular *E*. *coli* was measured using real-time PCR at the indicated times following infection of wt BMDMs with *E*. *coli* NC101 or *E*. *coli* O157:H7. Data are presented as means ± sd (n = at least 3 wells/timepoint, *p<0.05 vs. *E*. *coli* O157:H7).

Since *E*. *coli* O157:H7 are killed more efficiently by wt BMDMs than *E*. *coli* NC101 and since the *ibpAB-*mediated protection from intra-macrophage killing presumably requires adequate expression of *ibpAB*, we asked whether *E*. *coli* O157:H7 upregulate *ibpAB* after phagocytosis to a similar degree as *E*. *coli* NC101. To answer this question, we compared *ibpAB* expression in phagocytosed *E*. *coli* NC101 with *E*. *coli* O157:H7 in wt BMDMs. Although *E*. *coli* O157:H7 slightly increase i*bpAB* expression after infection of BMDMs, they do so to a much lesser extent compared with *E*. *coli* NC101 (Fig. 6). Therefore, it is conceivable that the increased killing of *E*. *coli* O157:H7 compared with *E*. *coli* NC101 by wt BMDMs may be due to insufficient *ibpAB* expression in *E*. *coli* O157:H7. These results support the concept that the *E*. *coli ibpAB* operon is a virulence factor that is upregulated in certain strains of *E*. *coli*, including NC101, during macrophage infection, and protects *E*. *coli* from killing by macrophage-derived ROS.

## Discussion

Several functions of *E*. *coli ibpAB* have previously been identified, including protection of bacteria from elevated temperatures, carbon monoxide, tellurite and copper toxicity, and oxidative stress[16–18,29,33]. However, all previously published studies have examined the roles of *ibpAB* in bacterial survival in laboratory cultures devoid of eukaryotic cells, and therefore have limited relevance to host-microbial interactions in animal systems. In our studies, we present new evidence that *ibpAB* also attenuate the bactericidal activity of macrophage ROS leading to increased survival of certain clinically-relevant *E*. *coli* strains within macrophages.

The mechanisms by which *ibpAB* protect *E*. *coli* from ROS are not entirely clear. The *ibpAB* gene sequences are not similar to those of known *E*. *coli* superoxide dismutases or catalase and therefore it is unlikely that IbpAB enzymatically neutralize superoxides and peroxides. More likely, IbpAB function as intracellular chaperones that bind and sequester or refold proteins that have been damaged by ROS, similar to the mechanisms by which they protect bacterial proteins from heat shock[28]. Indeed, others have shown that recombinant IbpA and IbpB suppress inactivation of *E*. *coli* metabolic enzymes by potassium superoxide and hydrogen peroxide in vitro and bind non-native forms of the enzymes[28]. Presumably, similar events occur within the cytoplasm of bacteria exposed to ROS or heat, but this concept remains to be proven.

Given that *ibpAB* protect *E*. *coli* proteins from damage by ROS, we hypothesized that *E*. *coli* upregulate *ibpAB* expression in response to ROS. In the present work, we show that ROS induce *ibpAB* expression in *E*. *coli* in lab cultures and macrophage phagolysosomes. Interestingly, while we detected a transient increase in *ibpAB* expression in *E*. *coli* cultures treated with the superoxide generator, paraquat, we did not detect upregulation of *ibpAB* in *E*. *coli* cultures treated with hydrogen peroxide (data not shown). The explanation for this difference is not entirely clear, but could be due to the more reactive and therefore damaging nature of superoxides compared with peroxides. We also hypothesized that RNS, like ROS, might induce *ibpAB* expression. However, contrary to our hypothesis, we observed increased *ibpAB* expression in *E*. *coli* within *Inos* ^*-/-*^ macrophages that are deficient in RNS production. This unexpected result could be due to compensatory upregulation of ROS production in *Inos* ^*-/-*^ macrophages, a phenonmenon that has previously been reported[34]. It is also notable that even in the *gp91phox* ^*-/-*^ macrophages that have impaired ROS production, *E*. *coli ibpAB* expression increases over time. Therefore, other factors within macrophages, besides ROS, likely play a role in *ibpAB* expression.

The mechanisms by which ROS cause transcription of *ibpAB* are unknown. Others have previously shown that the alternative sigma factors σ32 and σ54 transcribe *ibpAB* and ibpB, respectively[20]. In addition to heat, other factors have been shown to increase σ32 protein levels, including ethanol, hyperosmotic shock, carbon starvation, and alkaline pH. On the other hand, σ54 controls expression of several nitrogen-metabolism genes. However, changes in abundance or activity of these alternative sigma factors in response to oxidative stress have not been previously reported.

In addition to transcriptional control, IbpAB protein levels are also controlled at the levels of RNA processing, translation, and protein stability. [35,36]. In the present study, we show evidence suggesting that *ibpAB* expression is also controlled post-transcriptionally at the mRNA level. For instance, upregulation of *ibpAB* mRNA in *E*. *coli* treated with paraquat or phagocytosed by macrophages is partially dependent on the small regulatory RNA, *oxyS*. Our findings are somewhat surprising since a screen of mutants with a randomly inserted reporter gene failed to identify *ibpAB* as targets of regulation by *oxyS*[32]. In addition, *ibpAB* were not identified as putative targets of *oxyS* regulation using an in silico analysis[37]. Perhaps this discrepancy may be due to differences in assay design (e.g. reporter gene vs. real-time PCR) or false assumptions in computational prediction algorithms.

We have previously determined that colitis is associated with increased *ibpAB* mRNA levels in intra-colonic *E*. *coli*[23]. While our studies do not prove that ROS present at increased concentrations in inflamed colon tissue mediate the upregulation of *E*. *coli ibpAB*, they do demonstrate that *ibpAB* expression is at least partially induced by ROS in vitro and therefore suggest that ROS may contribute to *ibpAB* expression during colitis in vivo. Further studies in which colonic ROS are neutralized during colitis will be required to determine whether this is actually the case.

Since ROS cause *E*. *coli* to increase *ibpAB* expression and since *ibpAB* expression is associated with enhanced survival in BMDMs, one might predict that *ibpAB-*expressing *E*. *coli* are more virulent than *ibpAB-*deficient *E*. *coli* in diseases that are associated with persistence of bacteria within macrophages such as IBD’s and experimental colitis. On the contrary, we have previously shown that *ibpAB-*deficient *E*. *coli* paradoxically cause increased inflammatory responses in colitis-prone *Il10* ^*-/-*^ mice compared with wt mice by unknown mechanisms[23]. Therefore, the biological relevance of *ibpAB-*mediated increases in intra-macrophage *E*. *coli* survival that we observed in the present studies to experimental colitis is unclear. One possible explanation for the inverse relationship between intra-macrophage *E*. *coli* survival in these experiments and colitis severity in prior experiments is that macrophages used in the present study were obtained from C57/B6 mice whereas the colitis model requires the use of mice on the SvEv/129 genetic background. It is known that SvEv/129, but not C57/B6, mice are naturally deficient in the Slc11a1 (Nramp1) gene expressed in macrophages that functions to protect mice from certain intracellular bacterial infections[38,39]. Therefore, our findings in BMDMs from C57/B6 mice may not be applicable to Slc11a1-deficient SvEv/129 mice that have a baseline defect in killing of intracellular microbes. Nonetheless, we believe that our results highlight a potentially important pathway by which *E*. *coli* protect themselves from host immune responses.

In summary, we have identified a novel mechanism by which some *E*. *coli* increase transcription of *ibpAB* and have shown that the upregulation of *ibpAB* enhances survival of a non-pathogenic *E*. *coli* strain in macrophages. Further investigation of these proteins in other non-pathogenic and pathogenic bacterial strains in disease models will help clarify the role that they play as virulence factors in infectious and inflammatory disease pathogenesis.
